# Proteomics and Metabolomics Profiling of Platelets and Plasma Mediators of Thrombo-Inflammation in Gestational Hypertension and Preeclampsia

**DOI:** 10.3390/cells11081256

**Published:** 2022-04-07

**Authors:** Luiz Gustavo N. de Almeida, Daniel Young, Lorraine Chow, Joshua Nicholas, Adrienne Lee, Man-Chiu Poon, Antoine Dufour, Ejaife O. Agbani

**Affiliations:** 1 McCaig Institute for Bone and Joint Health, University of Calgary, Calgary, AB T2N 1N4, Canada; luizgustavo.almeida@ucalgary.ca (L.G.N.d.A.); daniel.young1@ucalgary.ca (D.Y.); antoine.dufour@ucalgary.ca (A.D.); 2 Department of Biochemistry and Molecular Biology, Cumming School of Medicine, University of Calgary, Calgary, AB T2N 1N4, Canada; 3 Department of Anaesthesiology, Perioperative and Pain Medicine, Cumming School of Medicine, University of Calgary, Calgary, AB T2N 1N4, Canada; lorraine.chow121@gmail.com (L.C.); jnichola@ucalgary.ca (J.N.); 4 Libin Cardiovascular Institute, Calgary, AB T2N 1N4, Canada; adrienne.lee@bccancer.bc.ca; 5 Division of Hematology, Department of Medicine/Medical Oncology, University of British Columbia, Island Health, Victoria, BC V6T 1Z4, Canada; 6 Division of Hematology & Hematological Malignancies, Department of Medicine, Cumming School of Medicine, University of Calgary, Calgary, AB T2N 1N4, Canada; mcpoon@ucalgary.ca; 7 Department of Physiology & Pharmacology, Cumming School of Medicine, University of Calgary, Calgary, AB T2N 1N4, Canada

**Keywords:** preeclampsia, gestational hypertension, pregnancy, proteomics, metabolomics, cytokine, chemokine, LRRC27/42, platelets

## Abstract

Platelets may be pivotal mediators of the thrombotic and coagulopathic complications of preeclampsia (PE), linking inflammation and thrombosis with endothelial and vascular dysfunction. Both PE and gestational hypertension (GH) fall within the spectrum of hypertensive complications of pregnancy, with GH being a risk factor for preeclampsia. However, it is unclear what biomarkers distinguish PE from GH. Using a discovery size cohort, we aimed to characterize specific plasma and platelet thrombo-inflammatory drivers indicative of PE and differentiate PE from GH. We performed multiplex immunoassays, platelet and plasma quantitative proteomics and metabolomics of PE patients, comparing with non-pregnant (NP), healthy pregnant controls (PC) and GH participants. The expression pattern of plasma proteins and metabolites in PE/GH platelets was distinct from that of NP and PC. Whilst procoagulation in PC may be fibrinogen driven, inter-alpha-trypsin inhibitors ITIH2 and ITIH3 are likely mediators of thrombo-inflammation in GH and PE, and fibronectin and S100A8/9 may be major procoagulant agonists in PE only. Also enriched in PE were CCL1 and CCL27 plasma cytokines, and the platelet leucine-rich repeat-containing protein 27 and 42 (LRRC27/42), whose effects on platelets were explored using STRING analysis. Through protein-protein interactions analysis, we generated a new hypothesis for platelets’ contribution to the thrombo-inflammatory states of preeclampsia.

## 1. Introduction

Preeclampsia (PE) complicates up to 8% of pregnancies; it is defined as elevated blood pressure (>140/90 mmHg on at least two occasions and >6 h apart) at or beyond 20 weeks gestation, and proteinuria (≥300 mg/24 h) or adverse conditions, such as elevated liver enzymes, which are known to increase the risk of severe complications [1,2]. Gestational hypertension (GH), considered a risk factor for PE [3,4,5], is characterized by an elevated blood pressure (sBP ≥ 140 mmHg and/or dBP ≥ 90 mmHg) at or beyond week 20 of pregnancy but without proteinuria or adverse conditions [1,2]. In this study, we used the definitions of hypertensive disorders of pregnancy according to the Society of Obstetricians and Gynecologists of Canada guidelines [1,6], to distinguish between preeclampsia and gestational hypertension. Both GH and PE are considered hypertensive disorders of pregnancy [7,8,9], with PE being more severe and resulting in more clinical complications than GH [7,8,9]. Preeclampsia can lead to significant adverse outcomes, including fetal and intrauterine growth restriction, severe maternal hypertension, and end-organ dysfunction that may result in preterm delivery, placental abruption, and maternal and perinatal death [1,10,11,12,13,14]. Its hematological manifestations include thrombocytopenia, thrombosis, and postpartum hemorrhage [1,6]. The cause of preeclampsia is still unclear, but there is evidence that platelets may be pivotal mediators of its complications, linking inflammation and thrombosis with endothelial and vascular dysfunction [15,16,17,18,19].

Early in pregnancy, platelets undergo differential granular secretions, releasing vasoactive agents important to the formation and maintenance of blood and lymphatic vessels, as well as to the maintenance of endothelial function and vascular integrity [17,20,21,22,23]. Procoagulant platelet factors are increased in patients with preeclampsia, such as plasma levels of transforming growth factor-β (TGF-β) and platelet factor-4 (PF4 or CXCL4) [18,22]. Additionally, several studies report on the role of micro- and nanoparticles present in the circulation in preeclampsia. When isolated and injected into pregnant mouse models, these particles can induce a preeclampsia state via direct effects on the endothelium to reduce proliferation and cause dysfunction [19,24]. The micro- and nanoparticles appear to originate mostly from activated platelets (97–99%), but they are also released from syncytiotrophoblasts [25]. It is well established that platelets are abnormally activated in preeclampsia [26,27], yet the precise agonist, or combination of agonists, at play in preeclampsia is unknown. Such a degree of procoagulation is not present in a healthy pregnancy. Taken together, the platelet activation process itself may contribute to the progression, if not the pathogenesis of preeclampsia.

Understanding the role of platelets in the development and or perpetuation of preeclampsia may enable the early identification of patients at risk of preeclampsia and the development of more targeted therapies. Besides having distinct epidemiology and hemodynamic characteristics, PE also differs from GH in pathology and pathogenesis [3,28,29,30,31,32]. In this study, we analyzed proteomics and metabolomics changes in plasma and platelets derived from PE and GH patients, compared with non-pregnant (NP) and healthy pregnant (PC) participants, to identify potential drivers of thrombo-inflammation in PE and GH. Next, we addressed whether PE can be distinguished from GH at the initial finding of hypertension by assessing biomarkers at time of diagnosis. Unique to PE was the overexpression in platelets of leucine-rich repeat-containing proteins 27/42 (LRRC27/42) [33,34], two proteins with limited characterization that could play biological roles in cell adhesion, signal transduction and apoptosis, and S100A9, a Ca^2+^ and Zn^2+^ protein regulating inflammation and immune responses [35,36], which we functionally validated. Lastly, we utilized pathway analysis to generate a new hypothesis for platelets’ contribution to the thrombo-inflammatory state of preeclampsia.

## 2. Methods

### 2.1. Methods Statement

Written informed consent was obtained in accordance with the Declaration of Helsinki. Blood samples were obtained from participants meeting study inclusion criteria (see Section 3) under the University of Calgary, Research Ethics Board approval (REB18-1545; online supplemental data). All methods were performed in accordance with the Alberta Health Services and The University of Calgary research guidelines and regulations.

### 2.2. Human Platelet-Rich Plasma Preparation

Blood was collected into blue-capped sodium citrate tubes (0.109 M/3.2%) and centrifuged at 180× *g* for 17 min to prepare platelet-rich plasma (PRP). All blood samples were processed within 1–2 h of collection.

### 2.3. Plasma and Platelet Lysates Preparation

PRP was centrifuged at 650× *g* to obtain the platelet-poor plasma (supernatant) for storage (−80 °C) until used. Plasma and platelets were lysed with buffer containing 1% SDS, 200 mM HEPES (pH 8.0), 100 mM ammonium bicarbonate, 10 mM EDTA and protease inhibitor cOmplete™ tablets (Roche, Mississauga, ON, Canada). Disulfide bonds were reduced with 10 mM Tris(2-carboxyethyl)phosphine hydrochloride (Thermo Fisher Scientific, Mississauga, ON, Canada) at 55 °C for 1 h and cysteines were alkylated with 15 mM iodoacetamide (VWR, Mississauga, ON, Canada) for 25 min in the dark at room temperature. Protein precipitation was performed with 600 μL of ice-cold acetone, incubated at −20 °C overnight, and followed with centrifugation at 8000× *g* for 10 min.

### 2.4. Quantitative Proteomics

The four groups of patients’ plasma and platelets were subjected to quantitative shotgun proteomics analysis, as previously described (Table 1) [37]. Plasma and platelet analysis were performed on non-pregnant (NP), healthy pregnant controls (PC), gestational hypertension (GH), and preeclampsia (PE) participants. A total of 100 μg per samples was analyzed. Proteins were resuspended in 100 μL of 50 mM triethyl ammonium bicarbonate and trypsinized (Thermo Fisher Scientific, Mississauga, ON, Canada) overnight at a 1:10 enzyme-to-substrate ratio. TMTsixplex™ (Thermo Fisher Scientific, Mississauga, ON, Canada) labeling was performed according to the manufacturer’s manual (Table S1). In summary, 0.8 mg of TMT reagent was resuspended in 41 μL of acetonitrile (ACN), samples were spun down at 4000× *g* for 10 s and incubated at room temperature for 1 h. The reaction was quenched with 8 μL of 5% hydroxylamine and incubated for 15 min at 25 °C. Peptides with different labels were combined followed by 100% formic acid (FA) addition to each sample to reach a volumetric concentration of 1% FA. Samples were spun at 5000 rpm for 10 min before desalting using Sep-Pak C18 columns (Waters, 130 mg WAT023501) conditioned with 1 × 3 mL 90% methanol/0.1% TFA, 1 × 2 mL 0.1% formic acid. Each sample was loaded onto a column, washed with 1 × 3 mL 0.1% TFA/5% methanol, and the peptides were eluted off the column with 1 × 1 mL 50% ACN/0.1% FA. Peptides were lyophilized prior to resuspension in 1% FA and a colorimetric peptide assay (Thermo Fisher Scientific, Mississauga, ON, Canada) was used to determine the concentration in each sample. The resulting tryptic peptide samples were dried down and stored at −80 °C.

### 2.5. High Performance Liquid Chromatography (HPLC) and Mass Spectrometry (MS)

Liquid chromatography and mass spectrometry experiments were carried out by the Southern Alberta Mass Spectrometry (SAMS) core facility at the University of Calgary, Canada. LC-MS/MS was performed on an Orbitrap Fusion Lumos Tribrid mass spectrometer (Thermo Fisher Scientific) operated with Xcalibur (version 4.0.21.10) and coupled to a Thermo Scientific Easy-nLC (nanoflow Liquid Chromatography) 1200 system. A total of 2 μg tryptic peptides per plex was loaded onto a C18 trap (75 um × 2 cm, Acclaim PepMap 100, Thermo Fisher Scientific, Mississauga, ON) at a flow rate of 2 μL/min of solvent A (0.1% formic acid and 3% acetonitrile in LC-MS grade water). Peptides were eluted using a 120 min gradient from 5 to 40% (5% to 28% in 105 min followed by an increase to 40% B in 15 min) of solvent B (0.1% formic acid in 80% LC-MS grade acetonitrile) at a flow rate of 0.3 μL/min and separated on a C18 analytical column (75 um × 50 cm, PepMap RSLC C18, Thermo Fisher Scientific, Mississauga, ON). Peptides were electrosprayed using 2.3 kV voltage into the ion transfer tube (300 °C) of the Orbitrap Lumos operating in positive mode. The Orbitrap first performed a full MS scan at 120,000 FWHM resolution to detect the precursor ion having a m/z between 375 and 1575 and a +2 to +4 charge. The Orbitrap Auto Gain Control (AGC) and the maximum injection time were set to 4 × 10^5^ and 50 ms, respectively. The Orbitrap was operated using the top speed mode with a 3 sec cycle time for precursor selection. The most intense precursor ions presenting a peptidic isotopic profile and having an intensity threshold of at least 2 × 10^4^ were isolated using the quadrupole (isolation window of m/z 0.7) and fragmented with HCD (38% collision energy) in the ion routing multipole. The fragment ions (MS2) were analyzed in the Orbitrap at a 15,000 resolution. The AGC, the maximum injection time, and the first mass were set at 1 × 10^5^, 105 ms, and 100, respectively. Dynamic exclusion was enabled for 45 s to avoid the acquisition of same precursor ion having a similar m/z (plus or minus 10 ppm).

### 2.6. Proteomic Data and Bioinformatics Analysis

Spectral data obtained during mass-spectrometry were matched to peptide sequences in the human protein database obtained from UniProtKB, containing reviewed, unreviewed, canonical and isoforms. The analysis was performed on MaxQuant [39] (v1.6.0.1) running the Andromeda algorithm [40] at a peptide-spectrum match FDR of <0.05. Search parameters included a mass tolerance of 20 p.p.m. for the parent ion, 0.5 Da for the fragment ion, fixed modification for carbamidomethylation of cysteine residues (+57.021464 Da), variable N-terminal modification by acetylation (+42.010565 Da), and variable methionine oxidation (+15.994915 Da). TMTsixplex™ labels 126 to 131 were defined as labels for relative quantification. The cleavage site specificity was set to Trypsin/P, with up to two missed cleavages allowed. Next, peptides and proteins from the evidence.txt and proteinGroups.txt files, respectively, were used for data analysis with MSstatsTMT (v1.8.2) [41] in R programming (v4.0.3) [42]. Default settings in MSstatsTMT were used for the analysis. For input conversion, “Gene.names” was used for protein ID, and the “msstats” method was selected for protein summarization. Significant outlier cut-off values were determined by interquartile boxplot analysis for each group comparison using the values of log2 fold-change [43].

### 2.7. Protein-Protein Interaction Analysis

The Search Tool for the Retrieval of Interacting Genes (STRING) database [44] was used to assess interconnectivity among proteins. For the analysis, proteins from the plasma and platelet proteomics were chosen based on the interquartile boxplot analysis and only outliers were selected. All the proteins from inflammatory mediators panel were selected. Proteins from the literature included solute carrier family 2—facilitated glucose transporter member 1 (SLC2A1, i.e.,: GLUT1), -3 (SLC2A3, i.e.,: GLUT3), aquaporin-1 (AQP1), piezo-type mechanosensitive ion channel component 1 (PIEZO-1), short transient receptor potential channel 6 (TRPC6), calcium-activated chloride channel regulator 1 (CLCA1), -2 (CLCA-2), -4 (CLCA-4), and platelet glycoprotein VI (GP6). The generated network was imported to Cytoscape (3.8.2) [45] for color coding each node and the addition of a border colored according to the log2 fold-change.

### 2.8. Metabolomics

Metabolomics runs were performed as shown previously [46] on a QExactive™ HF Hybrid Quadrupole-Orbitrap™ Mass Spectrometer coupled to a Vanquish™ UHPLC System. Chromatographic separation was achieved on a SyncronisHILIC UHPLC column (2.1 mm × 100 mm × 1.7 μm) using a binary solvent system at a flow rate of 600 μL/min. Solvent A, 20 mM ammonium formate pH 3.0 in mass spectrometry grade H_2_O; Solvent B, mass spectrometry grade acetonitrile with 0.1% formic acid (% *v*/*v*). The following gradients were used: 100% Solvent B (0–2 min), 100–80% Solvent B (2–7 min), 80–5% Solvent B (7–10 min), 5% Solvent B (10–12 min), 5–100% Solvent B (12–13 min), and 100% Solvent B (13–15 min). A sample injection volume of 2 μL was used. The mass spectrometer was run in negative full scan mode at a resolution of 240,000 scanning from 50 to 750 m/z. Metabolite analyses were completed using El Maven (v.0.12.0), a mass spectrometry data analysis software package [47,48]. Metabolites were identified by matching observed m/z signals (±10 p.p.m.) and chromatographic retention times to those observed from the reference Mass Spectrometry Metabolite Library.

### 2.9. Metabolomic Analysis

The metabolomics analysis was performed with MetaboAnalyst 5.0 [49]. Peak intensities were chosen as the data format. Data filtering was set to none, and the data transformation was set to log10. For the heatmap, the distance measure was set to Euclidean and the clustering method was set to ward. One-way analysis of variance (ANOVA) and Tukey’s HSD post-hoc analyses were used to determine proteins with statistically significant changes. For the enrichment analysis, we uploaded the data corresponding to the group comparison and selected quantitative enrichment with parameters set as categorical (classification) and compound names for metabolites. The data transformation was set to log10, and the metabolite library was set to Small Molecule Pathway Database (SMPDB), using only metabolite sets containing at least two entries. Lastly, the metabolite and protein ratios corresponding to a specific group comparison were uploaded for joint-pathway analysis. The organism was set to human, the metabolomics was set to targeted (compound list), gene list was set to official gene symbol and ID type was set to compound name. The pathway database, or universe, was set to metabolic pathways (integrated), with a hypergeometric test for the enrichment analysis, degree centrality for the topology measure, and combine queries for the integration method.

### 2.10. Multiplex Analysis of Human Cytokines

Plasma samples from healthy, non-diabetic, normotensive, non-pregnant controls (NP), normotensive pregnant controls (PC), gestational hypertensive controls (GH), or pregnant women with preeclampsia (PE) were analyzed for cytokines/chemokines as biomarkers of systemic inflammation. We used Luminex (Austin, TX, USA) xMAP technology for multiplexed quantification of 71 human cytokines, chemokines, and growth factors. The multiplexing analysis was performed using the Luminex™ 200 system (Luminex, Austin, TX, USA) by Eve Technologies Corp. (Calgary, AB, Canada). Seventy-one markers were simultaneously measured in the samples using Eve Technologies’ Human Cytokine 71-Plex Discovery Assay^®^, which consists of two separate kits: one 48-plex and one 23-plex (MilliporeSigma, Burlington, MA, USA). The assay was run according to the manufacturer’s protocol. The 48-plex consisted of: soluble cluster of differentiation 40 ligand (sCD40L); epidermal growth factor (EGF); eotaxin; basic fibroblast growth factor (FGF-2); FMS-like tyrosine kinase 3 ligand (FLT-3 Ligand); fractalkine; granulocyte colony-stimulating factor (G-CSF); granulocyte-macrophage colony-stimulating factor (GM-CSF); growth related oncogene alpha (GROα); interferon (IFN) alpha 2 and gamma (IFN-α2, IFN-γ); interleukin (IL) 1 alpha and 1 beta (IL-1α, IL-1β); interleukin 1 receptor antagonist (IL-1RA); interleukin (IL; IL-2, IL-3, IL-4, IL-5, IL-6, IL-7, IL-8, IL-9, IL-10; IL-12 subunit p40 and p70 (IL-12 [p40] and IL-12 [p70]); IL-13, IL-15; IL-17 isoform A, E, and F (IL-17A, IL-17E/IL-25, IL-17F); IL-18, IL-22, IL-27):, interferon gamma-induced protein 10 (IP-10); monocyte chemotactic protein 1 and 3 (MCP-1, MCP-3); macrophage colony-stimulating factor (M-CSF); macrophage-derived chemokine (MDC); monokine induced by interferon-gamma (MIG/CXCL9); marophage inflammatory protein 1 alpha and 1 beta (MIP-1α, MIP-1β); platelet-derived growth factor isoform AA and isoform AB/BB (PGF-AA, PDGF-AB/BB); regulated on activation, normal T Cell expressed and secreted (RANTES); transforming growth factor alpha (TGFα); tumor necrosis factor alpha and beta (TNF-α, TNF-β); and vascular endothelial growth factor A (VEGF-A). The 23-plex consisted of: chemokine [C-C motif] ligand 21 (6CKine); B cell-attracting chemokine 1 (BCA-1); cutaneous T cell-attracting chemokine (CTACK); epithelial neutrophil-activating peptide 78 (ENA-78); eotaxin-2; eotaxin-3; chemokine [C-C motif] ligand 1 (I-309); interleukin (IL) 16, IL-20, IL-21, IL-23; IL-28 isoform A (IL-28A); IL-33; leukaemia inhibitory factor (LIF); monocyte chemotactic protein 2 and 4 (MCP-2, MCP-4); macrophage inflammatory protein 1 delta (MIP-1δ); stem cell factor (SCF); stromal-derived factor 1 alpha + beta (SDF-1α + β); thymus- and activation-regulated chemokine (TARC); thrombopoietin (TPO); tumor necrosis factor-related apoptosis-inducing ligand (TRAIL); and thymic stromal lymphopoietin (TSLP). Assay sensitivities of these markers range from 0.14–55.8 pg/mL for the 71-plex. Individual analyte sensitivity values are available in the MILLIPLEX protocol.

### 2.11. Platelet Procoagulant Membrane Dynamics Imaging

Glass-bottom MatTek dishes were pre-coated with bovine serum albumin (BSA). Aliquots of platelet rich plasma (PRP) recalcified to 1 mmol/L with CaCl_2_ were pre-incubated (15 min) with probes (as indicated in figures and legends) and added onto MatTek dishes. Adherent plasma platelets were fixed with 4% paraformaldehyde for 15 min after 45 min incubation. High-resolution 3D fluorescent images of platelets adherent over BSA surfaces were obtained at 25 °C using a confocal microscope. Image resolution was improved by the restoration complement of Volocity^®^ imaging Software Suite and analyzed using the same software (Quorum Technologies Inc., Puslinch, ON, Canada).

### 2.12. Statistical Analysis

Cytokine panel statistics was done using Prism 9.3 (GraphPad Software). No statistical methods were used to predetermine sample sizes. Individual analyses are reported in figure legends. Significantly changing proteins, from the proteomics data, were determined as the outliers after BoxplotR analysis. Outliers were determined as per Tukey’s definition [50].

### 2.13. Data and Code Availability

The proteomics data are publicly available and were deposited in PRIDE Archives, accession number: PXD031278. The R codes are available upon request.

## 3. Results

### 3.1. Study Participants

Study participants were either non-pregnant (NP) or patients with preeclampsia (PE), gestational hypertension (GH), or healthy pregnant controls (PC). All participants were non-diabetic, and patients showed no clinical signs of thrombotic events up to 48 h after delivery. Participant’s demographics, anthropometrics and platelet indices are shown in Table 1 [1,38]. We measured ‘Hemoglobin’ and ‘Platelet Indices’ in all study participants. Data were tested for normality using the Shapiro–Wilk test (*p* < 0.05) and analyzed with GraphPad Prism 9 (San Diego, CA, USA). Statistical tests was by 1-way analysis of variance (ANOVA) between groups or the Kruskal–Wallis test for between group differences, and *p* < 0.05 (*) or *p* < 0.01 (**) were considered significant. A post-hoc pairwise independent samples *t*-test yielded an insignificant *p*-value for test groups compared to healthy pregnant controls (Table 1).

### 3.2. Plasma Proteomics Analysis

**Figure 1:**
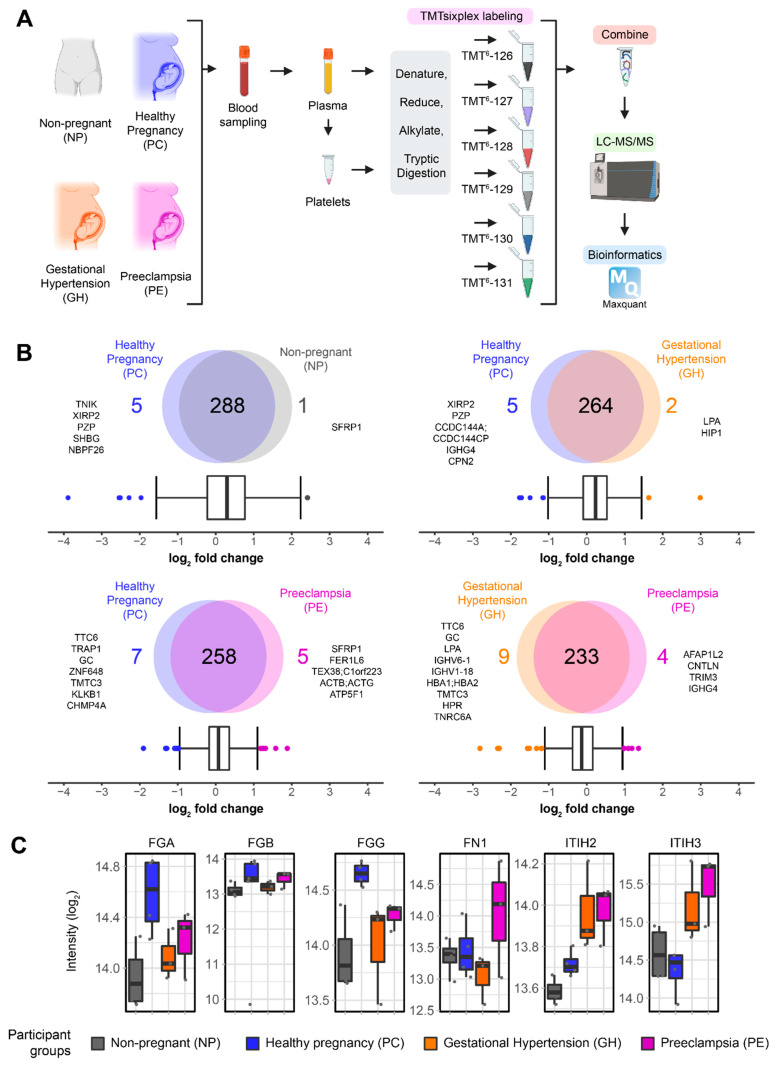
(**A**) Schematic of the proteomics workflow of non-pregnant (NP), healthy pregnancy (PC), gestational hypertension (GH), and preeclampsia (PE) patients. The plasma and platelets were extracted and submitted to a TMTsixplex labeling and shotgun proteomics workflow. (**B**) Quantification results of plasma proteomics and multiple group comparison as illustrated by Venn Diagrams. Interquartile boxplot analysis was performed to identify the differently expressed proteins as represented by outliers. (**C**) Fibrinogen alpha chain (FGA), fibrinogen beta chain (FGB), fibrinogen gamma chain (FGG), fibronectin 1 (FN1), inter-alpha-trypsin inhibitor heavy chain H1 (ITIH2), and -3 (ITIH3) intensity as quantified by shotgun proteomics. Data are represented as boxplots.

To identify differences between NP, PC, GH and PE, we performed quantitative shotgun proteomics of plasma participant samples. Plasma and platelet lysate proteins were labeled using TMTsixplex^TM^ followed by multiplexing for quantitative analysis (Figure 1A, Table S1). Using Maxquant [39], we identified a total of 6870 peptides corresponding to 391 proteins (Tables S2 and S3). We removed possible contaminants, mismatches, and proteins with less than two quantification values using MSstatsTMT and R programming, reaching a final count of 292 proteins (Table S4). Using quantitative plasma proteomics, multiple proteins were identified to be significantly changing between preeclampsia (PE) and gestational hypertension (GH); and between PE and healthy pregnancy (PC). Multiple group comparisons were performed according to their biological relevance (Figure 1B). To identify proteins that were significantly changing, we used the interquartile boxplot analysis [43], a descriptive statistical approach that identified outliers in the dataset. For non-pregnant (NP) vs. healthy pregnancy (PC), we identified six proteins changing, with one changing in NP (secreted frizzled-related protein 1, SFRP1) and five in PC (Figure 1B). Seven changing proteins were observed for gestational hypertension (GH) vs. healthy pregnancy (PC), in which two proteins were enriched in GH and five were enriched in PC. An increase in the number of changing proteins to 12 was observed when comparing preeclampsia (PE) vs. healthy pregnancy (PC), where five proteins were enriched in PE and seven proteins were enriched in PC. An even higher number of changing proteins (13) was observed when comparing preeclampsia (PE) vs. gestational hypertension (GH), with four proteins enriched in PE and nine proteins enriched in GH. Besides the protein changes evidenced by the boxplot analysis, we also observed notable differences in proteins known to be associated with a preeclampsia phenotype, as has been reviewed elsewhere [51]. Among these proteins, we highlight the differences in fibrinogen subunits alpha (FGA), beta (FGB), and gamma (FGG), fibronectin 1 (FN1), and inter-alpha-trypsin inhibitor heavy chain H2 (ITIH2) and -4 (ITIH3) (Figure 1C). Interestingly, hierarchical clustering and heatmaps of the quantified proteins grouped the three fibrinogen subunits along with other components at high expression in the pregnant controls (PC) (Figure 1C and Figure S1). Similarly, FN1, ITIH2 and ITIH3 grouped with proteins at relatively low expression in NP and PC groups but at higher levels in GH and PE.

### 3.3. Multiplex Immunoassays

**Figure 2:**
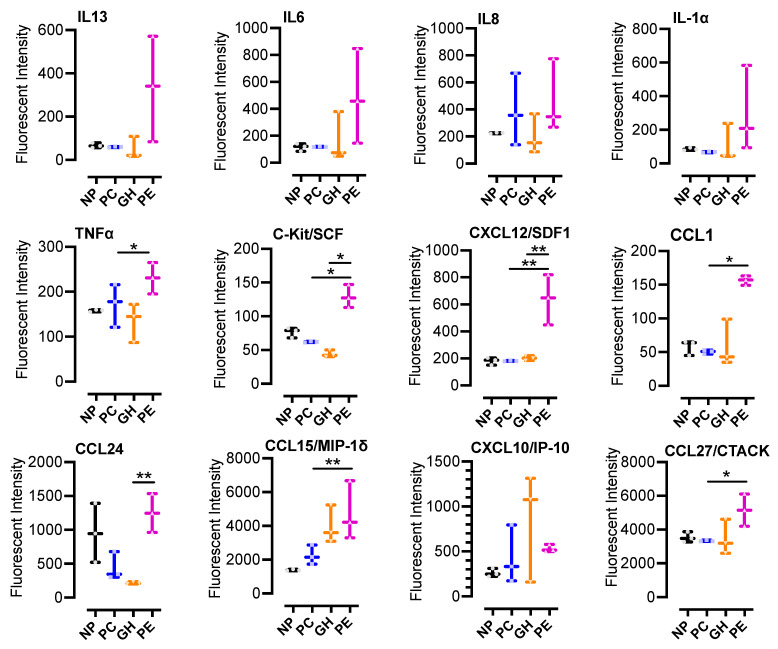
Multiplex immunoassay indicates the elevation of plasma CCL1 and CCL27, and multiple cytokines in preeclamptic patients: plasma samples (*n* = 3, per group) from non-pregnant (NP), healthy pregnant or pregnant controls (PC), gestational hypertensive (GH) and preeclamptic (PE) participants were analyzed for the indicated cytokine/chemokine as biomarkers of systemic inflammation. The multiplex immunoassay was performed with the Millipore MILLIPLEX Cytokine Array Assay Kit, and the results were analyzed with a Bio-Plex 200. Sample analysis was performed in duplicates for each participant, using the Bio-Plex Multiplex System, powered by Luminex xMAP technology. Data were then analyzed using GraphPad Prism 9 and were summarized in box-and-whisker plots for each biomarker. The plots show minimum to maximum values and dots/points represent all raw data (replicate experiments inclusive). The medians and interquartile ranges of data are represented by the horizontal line through the box and width of the box, respectively. Statistical significance was determined by 1-way ANOVA and Bonferroni adjusted post hoc independent samples *t*-tests. *p* < 0.05 (*) or *p* < 0.01 (**) were considered significant.

To identify differentially expressed plasma chemokines and cytokines, we performed a multiplex immunoassay. TNFα, C-Kit/SCF, CXCL12/SDF1, and CCL1 were significantly increased in PE but not in NP, PC or GH (Figure 2). Additionally, CCL15/MIP-1δ and CCL27/CTACK were statistically increased in PE when compared to PC (Figure 2). Our data demonstrate multiple chemokines and cytokines significantly elevated in PE but not in GH or PC, suggesting increased inflammation in the PE status. Furthermore, our analysis confirmed previous reports of increases in plasma cytokines, including CXCL12/SDF-1α, known to be able to directly activate platelets [52].

### 3.4. Platelets Proteomics Analysis

**Figure 3:**
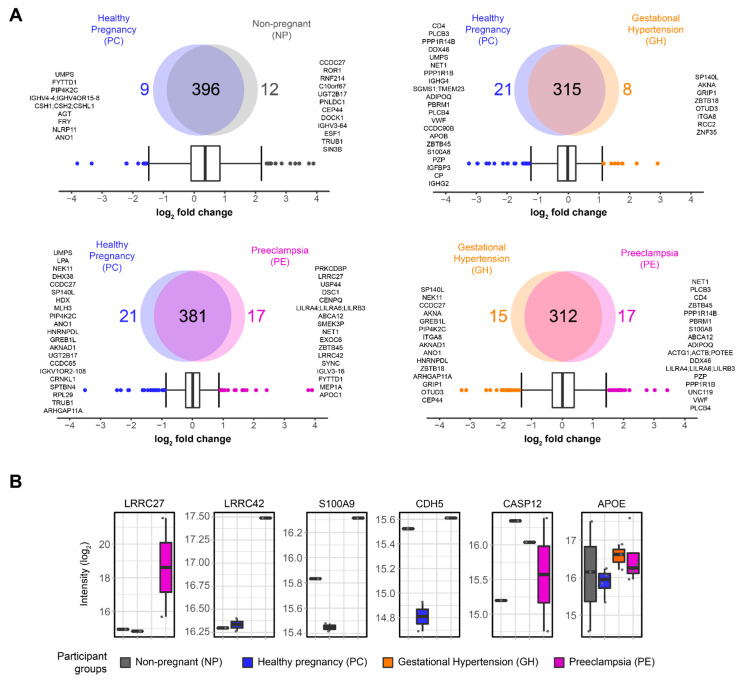
Platelet proteomics of non-pregnant (NP), healthy pregnancy (PC), gestational hypertension (GH), and preeclampsia (PE) patients. (**A**) Quantification results and multiple group comparison as illustrated by Venn Diagrams. Interquartile boxplot analysis was performed to identify the differently expressed proteins as represented by outliers. (**B**) Leucine-rich repeat-containing protein 27 (LRRC27) and -42 (LRRC42), and the top changing protein associated with inflammation (GO:0006954): protein S100A9 (S100A9), Cadherin-5 (CDH5), Caspase-12 (CASP12), and apolipoprotein E (APOE). Intensity was quantified by shotgun proteomics. Data are represented as boxplots.

We next performed a quantitative proteomics approach on platelet lysate derived from our study participants (Figure 1A). Platelet-derived drivers of thrombosis or inflammation may be diagnostic markers of PE, resulting in a molecular separation from GH [53]. Using Maxquant [39], we identified a total of 15,334 peptides corresponding to 592 proteins (Tables S5 and S6). An increase in the number of quantified proteins is likely observed due to the reduction in plasma contents during the enrichment phase. Plasma contains highly concentrated proteins, such as albumin, that often dominate the spectra obtained during data acquisition on the mass spectrometer, thus making it challenging to quantify proteins with low abundances, such as those present in the platelets [54]. After data processing via MSstatsTMT and R programming, a total of 419 proteins remained (Supplementary Table S7). Next, we applied an interquartile boxplot analysis on the comparisons with biological relevance (Figure 3A). For non-pregnant (NP) vs. healthy pregnancy (PC), we identified 12 proteins enriched in NP and 9 enriched in PC. The comparison of gestational hypertension (GH) vs. healthy pregnancy (PC) showed enrichment of eight proteins in GH and twenty-one enriched in PC. Interestingly, preeclampsia (PE) vs. healthy pregnancy (PC) also revealed 21 proteins enriched in PC; however, PE was enriched for 17 proteins, almost two-fold compared to GH vs. PC, possibly indicating broad disturbances in the blood of preeclampsia patients. Lastly, the comparison of preeclampsia (PE) vs. gestational hypertension (GH) showed enrichment of 17 proteins in PE and 15 proteins in GH. Inflammation is elevated in preeclampsia patients [55,56]; therefore, we investigated what inflammatory proteins were significantly changing in our data. We used PE vs. PC as our dataset of reference and queried all the proteins against the Gene Ontology for inflammation (GO:0006954). We found a total of 46 proteins, where the absolute log_2_ fold change indicated protein S100A9 (S100A9), cadherin-5 (CDH5), caspase-12 (CASP12), fibronectin (FN1), and apolipoprotein E (APOE) as the top five most changing proteins (Figure 3B). In addition to the inflammatory proteins, we also explored the differences in expression for leucine-rich repeat-containing protein 27 (LRRC27) and -42 (LRRC42), as they were indicated by the boxplot analysis as being significantly changing, while their role in platelets is largely unknown (Figure 3B).

### 3.5. Recombinant Human S100A8/S100A9 Heterodimer Induced Platelet P-Selectin Membrane Expression, Integrin Activation and Clumping In Vitro

**Figure 4:**
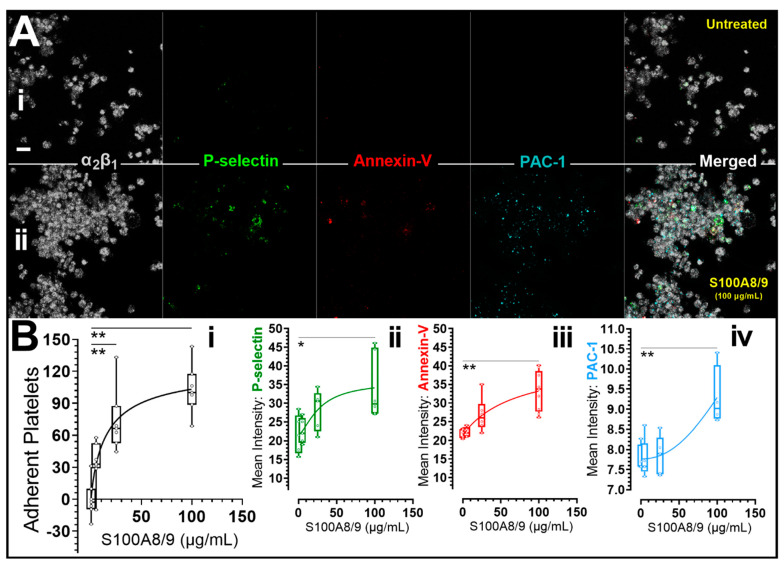
Recombinant human S100A8/S100A9 heterodimer induced platelet P-selectin membrane expression, integrin activation and clumping in vitro. Citrated platelet-rich plasma (PRP) from non-pregnant participants was untreated or preincubated with recombinant human S100A8/S100A9 (Catalog # 8226S8, Bio-Techne Canada, Toronto, ON, Canada) and labelled with human integrin α_2_/CD49b AlexaFluor^®^ 405-conjugated Antibody (Grey), AlexaFluor^®^ 488 anti-human CD62P (P-Selectin) antibody (Green), annexin V, AlexaFluor^®^ 568 conjugate (Red) and Alexa Fluor^®^ 647 anti-human CD41/CD61 (PAC-1) antibody (Cyan). PRP was then recalcified to 1 mM calcium and allowed to adhere (45 min) to surfaces precoated with bovine serum albumin (BSA). Image panel A shows representative extended focus view of untreated (**Ai**) and 100 ug/mL S100A8/S100A9 treated (**Aii**) platelets adherent to BSA surfaces. In panel (**Bi**–**Biv**), the mean fluorescent signal intensities in pooled experiments are shown. Data were tested for normality using the Shapiro-Wilk test (*p* < 0.05). Data were then analyzed using GraphPad Prism 9.3 (San Diego, CA) and presented as box-and-whiskers plots showing minimum to maximum values. The dots/points in the chart represent raw data (replicate experiments inclusive). The medians and interquartile ranges of data are represented by the horizontal line through the box and height of the box, respectively. Statistical significance was determined by 1-way ANOVA and Bonferroni post hoc tests. *p* < 0.05 (*) or *p* < 0.01 (**) were considered significant. Images were captured at Nyquist using a Nikon A1R laser scanning confocal microscope (original objective magnification, ×63) and analyzed using Volocity^®^ Software (Quorum Technologies, Lewes, UK). Scale bars: 5 μm.

S100A8/A9 has been previously associated with a predisposition to cardiovascular diseases [36,57], metabolic inflammation [58] and changes in platelet reactivity [59]. Therefore, we utilized recombinant human S100A8/A9 to validate the potential effects of S100A9 on the function of plasma platelets of our non-pregnant participant. Platelet degranulation was assessed by quantifying fluorescence intensity associated with alpha granule P-selectin (CD62P) membrane expression. Platelet phosphatidylserine (PS) exposure and integrin a_IIb_β_3_ activation were monitored using a conjugated annexin-V and PAC-1 antibody. As shown in Figure 4, we identified an increased P-selectin expression, PS exposure, α_IIb_β_3_ activation and platelet clumping after platelet treatment with 100 μg/mL recombinant S100A8/A9.

### 3.6. Protein-Protein Interaction Analysis

**Figure 5:**
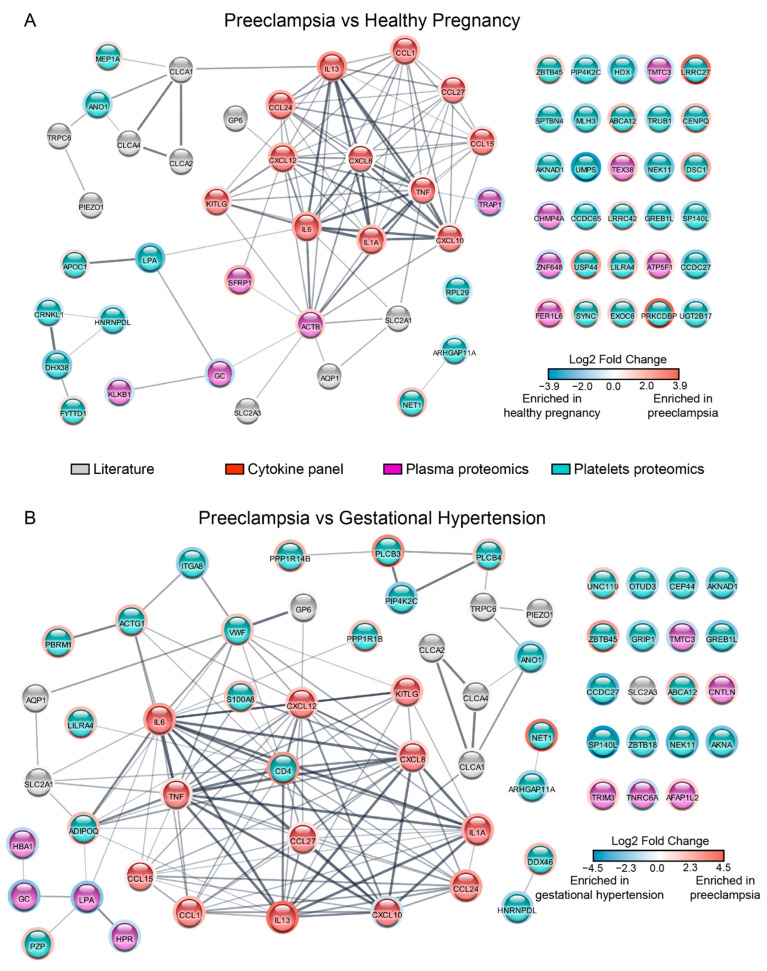
Integrative analysis represented by protein-protein interaction network. Preeclampsia vs. healthy pregnancy (**A**), and preeclampsia vs. gestational hypertension (**B**). Plasma and platelet proteomics are represented by differently expressed proteins as determined by boxplot. All cytokines and chemokines were included. Additional proteins were selected from the literature, including solute carrier family 2—facilitated glucose transporter member 1 (SLC2A1, i.e.,: GLUT1), -3 (SLC2A3, i.e.,: GLUT3), aquaporin-1 (AQP1), piezo-type mechanosensitive ion channel component 1 (PIEZO-1), short transient receptor potential channel 6 (TRPC6), Calcium-activated chloride channel regulator 1 (CLCA1), -2 (CLCA-2), -4 (CLCA-4), and platelet glycoprotein VI (GP6). Each node was colored according to its data source: literature = grey, cytokine and chemokines = red, plasma proteomics = magenta, and platelets proteomics = teal. The border color of each node changes according to the fold change; red is enriched in preeclampsia, and blue is enriched in either healthy pregnancy (**A**), or gestational hypertension (**B**).

To further identify distinct enriched or dysregulated biological functions in preeclampsia and gestational hypertension, we used the Search Tool for the Retrieval of Interacting Genes (STRING) database to map the protein-protein interactions identified in our dataset (Figure 5). We submitted a list of changing proteins, according to the boxplot analysis, along with the proteins assayed in the cytokine panel and additional proteins from the literature. By comparing preeclampsia and healthy pregnancy (Figure 5A), we identified a moderately-connected network, pointing to some crosstalk between the plasma and the platelets proteomics. Importantly, a lack of connections does not imply that protein-protein interaction does not exist; instead, it indicates that possible links are missing in the literature and remain to be explored. This is the case for leucine-rich repeat-containing protein 27 (LRRC27) and -42 (LRRC42), which are upregulated in the proteomics data, but little is known about both proteins. Next, we submitted the preeclampsia and gestational hypertension comparison for STRING analysis, leading to a well-connected network with multiple crosstalk between the plasma and the platelets. The proteins selected from the literature are also embedded in the network, indicating that the changes observed on our proteomics dataset possibly translate to downstream signaling that was not captured in our study (Figure 5B).

### 3.7. Metabolomics Analysis

**Figure 6:**
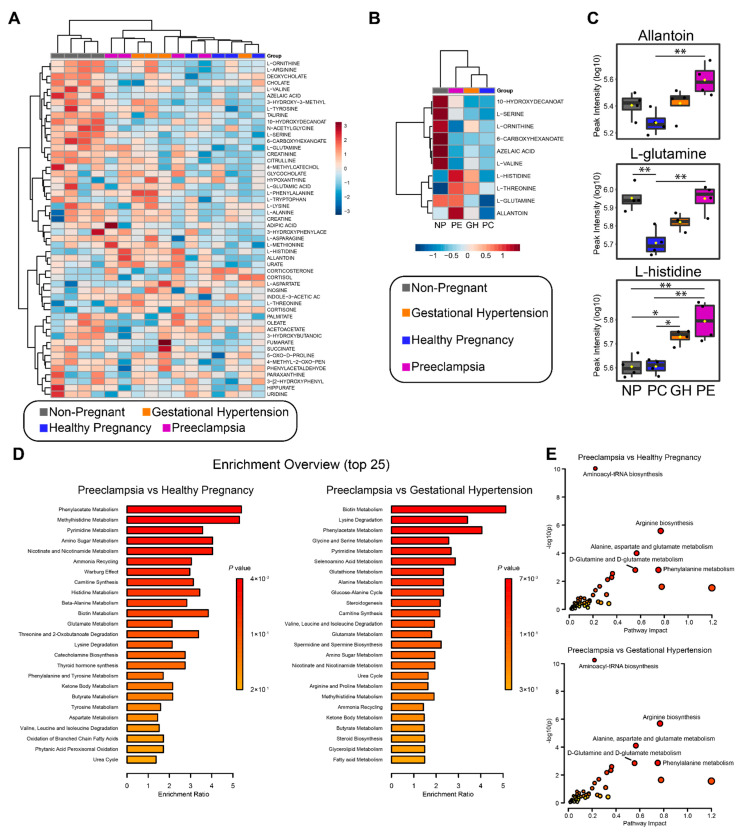
Metabolomics analysis from non-pregnant (NP), healthy pregnancy (PC), gestational hypertension (GH), and preeclampsia (PE) patients. Hierarchical clustering and heatmap of all the identified metabolites per sample (**A**) and the statistically changing metabolites as determined by ANOVA and Tukey’s HSD (**B**). Distance measure = euclidean and clustering algorithm = ward.D. (**C**) Allantoin, L-glutamine, and L-histidine intensity as determined by LC-MS and represented by boxplot. Statistical analysis performed with ANOVA and Tukey’s HSD. *p* < 0.05 (*) or *p* < 0.01 (**) were considered significant. (**D**) Quantitative enrichment analysis (QEA) of all the identified metabolites. Enrichment ratio was computed by hits/expected (hits = observed hits; expected = expected hits). (**E**) Joint-pathway analysis of the metabolomics and proteomics data. All the proteins and metabolites identified by each methodology were submitted to the analysis, along with their fold-change. Enrichment was performed via hypergeometric test, topology measure was set to degree centrality, and combined queries was the integration method.

To better characterize these clinical conditions and to further explore the phenotype observed in preeclampsia patients, we performed plasma metabolomics of our NP, PC, GH and PE participants (Figure 6, Table S8). We identified a total of 52 metabolites (Figure 6A). Using Metaboanalyst [49], we identified 10 metabolites statistically changing, along with a hierarchical clustering that clearly separates NP from PC, GH, and PE (Figure 6A,B). We identified allantoin, L-glutamine and L-histidine to be significantly elevated in PE or PE and GH when compared to PC (Figure 6C); specifically, allantoin was significantly elevated in PE compared to PC or GH. Also, L-glutamine was elevated in PE compared to GH or PC but not NP, and L-histidine was significantly elevated in PE and GH when compared to PC or NP (Figure 6C). Next, we performed pathway enrichment analysis using Metaboanalyst to identify patterns that are biologically meaningful and enriched in the metabolomics data. For PE vs. PC, we found significant enrichment of phenylacetate metabolism, methylhistidine metabolism, and pyrimidine metabolism, among others (Figure 6D). For PE vs. GH, the top three enriched pathways included biotin metabolism, lysine degradation, and phenylacetate metabolism (Figure 6D). Lastly, we sought to integrate the metabolomics and the proteomics data by running an integrated analysis in Metaboanalyst. We found a profile that was largely repeated on both PE vs. PC and PE vs. GH. Overall, there was strong enrichment for aminoacyl-tRNA biosynthesis, arginine biosynthesis, alanine-aspartate-glutamate metabolism, D-glutamine and D-glutamate metabolism, and phenylalanine metabolism (Figure 6E).

## 4. Discussion

Platelets undergo functional changes during a healthy pregnancy that indicate a gradual inherent activation as platelets become more sensitive to stimulation and primed to aggregate and adhere readily to the subendothelial matrix. This state of primed and activated platelets contributes to the gestational thrombocytopenia seen in healthy pregnancy and usually do not require treatment alterations [60]. Activated platelets can aggregate and adhere in circulation resulting in increased clearance [61]. This leads to increased platelet turnover and can result in the higher immature platelet fraction (IPF) with increased mean platelet volume (MPV) that accompanies the thrombocytopenia in healthy pregnancies. However, pathologic exacerbation of this innate platelet activation in GH and PE may account for the higher MPV and IPF seen in our small cohort, and as reported by others [53,62,63].

While this platelet priming is desirable for pregnant women to meet the haemostatic challenges of childbirth, the tendency towards procoagulation/thrombosis is increased in gestational hypertension and even more so in preeclampsia [26,64,65]. Our discovery-size cohort proteomics analysis confirmed previous findings of procoagulation mediators in healthy pregnancy (PC) and preeclampsia (PE) [51,66]. In addition, we reveal plasma mediators of procoagulation in gestational hypertension. Whilst several publications have reported findings on the plasma and serum proteomics of preeclampsia patients [51,66,67], nearly all have compared their findings with healthy pregnant control cohorts without clinical symptoms of hypertension, despite gestational hypertension being a major risk factor for preeclampsia. Therefore, our study fills a critical gap in the literature by comparing plasma and platelet proteomics data in preeclampsia with healthy pregnancy and gestational hypertension controls. Roles in blood coagulation and protein homeostasis during pregnancy have been reported for plasma kallikrein (KLKB1) [68], and pregnancy zone protein (PZP) [69], respectively. However, the roles of other proteins elevated in healthy pregnancy (Figure 1B), may be unknown or unlinked to coagulation. For example, XIRP2 (protects actin filament depolymerization); CCDC144A (plays a role in preventing the formation of kidney stones through inhibition of calcium oxalate monohydrate crystallization); IGHG4 (an immunoglobulin with no established link to coagulation); CPN2 (implicated in the complement activation pathway); TTC6 (unknown role); TRAP1 (involved in maintaining mitochondrial function and polarization); GC (involved with Complement 5); ZNF648 (a zinc finger involved in transcription); TMTC3 (an O-mannosyl-transferases); CHMP4A (a probable core component of the endosomal sorting required for transport complex III (ESCRT-III) that is involved in multivesicular bodies (MVBs) formation and sorting of endosomal cargo proteins into MVBs) [70,71]. In Figure 1B and Tables S2–S4, we showed all plasma proteins alterations in our study that were significant for each group comparison. Whereas the plasma of our PC participants was enriched in fibrinogen as previously reported [51,66], increased plasma inter-alpha-trypsin inhibitors ITIH2 and ITIH3 distinguished our GH and PE from PC participants, and plasma fibronectin may be a distinct driver of the heightened procoagulation in preeclampsia (Figure 1C).

The inter-alpha-trypsin inhibitors ITIH2 and ITIH3 are part of a family of structurally related plasma serine protease inhibitors (ITIH1-5) and have been reported to contribute to extracellular matrix stability by covalent linkage to hyaluronan [72,73]. There is a paucity of data on the role of ITIH2 and ITIH3 in platelet function and thrombosis. However, these proteins have been shown to play significant role in inflammation and in the prevention of cancer metastasis [74,75,76]. In this study, the enrichment of ITIH2 and ITIH3 in the hypertensive pregnancies (GH, PE) was distinguished from the relatively low expression in NP and PC statuses. ITIH proteins have previously been linked to inflammation [75,76,77], suggesting the role of these inflammatory cytokines in hypertensive pregnancies. Plasma ITIH may therefore be a biomarker of inflammation useful for monitoring the onset and progression of gestational hypertension and preeclampsia.

Pregnancy is a hypercoagulable physiological state driven by changes in plasma levels of blood coagulation and fibrinolytic factors. Our plasma proteomics profiling confirmed the phenotypic observation of increased plasma fibrinogen during healthy pregnancy when compared to the matched non-pregnant population (Figure 1B). Levels of fibrinogen in the non-pregnant population range from 360–530 mg/dl, and it may reach up to 600 mg/dL during healthy pregnancy [78,79]. Enhanced fibrinogen binding to activated platelets has been shown to correlate with the progression of healthy pregnancy to preeclampsia [64,65]. Thus, elevated fibrinogen levels alone may just be more indicative of the pregnant state [64], and a preponderance of activated platelets is required to shift this physiological priming to the pathological prothrombotic state of preeclampsia [26,64,65]. Interestingly, the increased level of plasma fibronectin in PE may serve as a biomarker of this disorder. Furthermore, plasma fibronectin levels may distinguish preeclampsia from gestational hypertension. Consistent with our findings, an earlier study of 33 preeclampsia patients and 26 healthy pregnant controls showed an increased level of fibronectin, which correlated with the evidence of endothelial injury [80]; and both endothelial injury and fibronectin level resolved after delivery [80]. Fibronectin is a ligand of platelet surface receptors and also an extracellular matrix component [81]. Recent studies have demonstrated that fibronectin may have procoagulant properties, driving platelet activation via the platelet collagen receptor glycoprotein (GP) VI and resulting in increased procoagulant activity and blood clotting [82,83,84,85,86]. Moreover, plasma fibronectin concentration has been shown to be a determinant of thrombus formation in models of in vivo thrombosis [82,83]. In addition, fibrillar cellular fibronectin has been shown to constitute a thrombogenic surface that induced strong platelet activation, platelet aggregation and procoagulant activity via α5β1 and αIIbβ3, the GPIb-V-IX complex, and GPVI [84]. Plasma fibronectin may also be vital to control bleeding in fibrinogen-deficient or anticoagulated states [85]. Taken together, platelets in preeclampsia are extensively activated when in circulation [27], and the distinctive increase in plasma fibronectin under this condition is consistent with an augmented prothrombotic state. Our data indicate that women at risk of developing preeclampsia could carry activated platelets, co-stimulated at least by plasma fibronectin. In addition, our data indicate that plasma fibronectin levels may serve as a prognostic biomarker in detecting preeclampsia when evaluating hypertensive disorders of pregnancies [80].

In preeclampsia, it is thought that vasoactive factors released from the placenta alter plasma levels of angiogenic factors and pro-inflammatory cytokines to trigger an exaggerated inflammatory response and endothelial cell dysfunction [87,88]. In addition, studies have implicated chemokines as mediators of several biological changes, including angiogenesis, chronic inflammation, fibro-proliferative disorders and preeclampsia [89]. Our multiplex immunoassays (Figure 2) confirmed previous reports in preeclampsia of increases in plasma cytokines, including angiostatic CXCL10 and CXCL12/SDF-1α [87]. Whilst plasma levels of CXCL10 were comparable between our PC, GH, and PE study participants, CXCL12/SDF-1α was significantly increased in PE patients by two-fold or more (Figure 2) when compared to PC or GH patients. It is believed that dysregulation of placental derived vasculogenic and angiogenic substances in maternal blood, such as stromal cell-derived factor-1 (SDF-1/CXCL12) underlie the pathogenesis of preeclampsia [90,91]. Increases in maternal blood CXCL12 have been reported in preeclampsia [92], and correlated with increased syncytiotrophoblast CXCL12 staining [92] and intrauterine growth restriction [93]. Since CXCL12 is a chemokine known to also activate platelets [52], preeclampsia onset and progression may correlate with plasma CXCL12 levels and the extent of activated platelets in maternal circulation. Therefore, plasma CXCL12 monitoring may identify pregnancies at higher risk of progression to pre/eclampsia. We also showed that plasma levels of CCL15/MIP-1δ and CCL27/CTACK increased in the order of NP < PC < GH < PE (Figure 2). Therefore, their levels may quantitatively correlate with progression from healthy pregnancy to preeclampsia.

We identified elevated levels of S100A9 in lysates of preeclampsia platelets (Figure 3). S100A8 and S100A9 are Ca^2+^ binding proteins constitutively expressed in neutrophils and monocytes and function as Ca^2+^ sensors [58]. During inflammation, S100A8/A9 is released to modulate the inflammatory response by stimulating leukocyte recruitment and cytokine secretion [58]. Furthermore, the expression levels of S100A8 in the peripheral blood of PE patients has been correlated positively with TNF-α, IL-6, and IL-12, but negatively with IL-10 [94]. Platelets express S100A9 [95,96]. Agonist stimulation has been shown to increase platelet mobilization and membrane expression of S100A9 from intracellular location [95]. Importantly, a causal role for S100A9 in thrombosis has been reported using multiple models of vascular injury [95]. Furthermore, in our validation experiment, stimulating platelets of our non-pregnant participants with recombinant S100A8/9 resulted in an increase in platelet microaggregation and clumping in a concentration-dependent manner and in association with integrin α_IIb_β_3_ activation as determined by PAC-1 binding (Figure 4(Ai,Aii,Bi–Biv)). At the same time, platelets treated with S100A8/9 showed a mild increase in α-granule degranulation and procoagulant activity. Platelets may incorporate S100A8 in S100A8/A9 heterodimers released from neutrophils [59]. Additionally, platelet derived S100A9 has been shown to directly modulate platelet function and thrombosis, and the mechanism is thought to be platelet CD36 dependent [95]. Therefore, it seems plausible that increased plasma S100A8/9 arising from leucocytes or activated platelets can contribute to platelet microaggregation in preeclampsia [97]. Further research is needed in this area. We speculate that S100A8/9 may be a target for antithrombotic treatment and could be used as a biomarker of preeclampsia and as an indicator of response to treatment. Taken together, the potential roles of S100A8/9 and CXCL12/SDF-1α in thrombo-inflammation represent examples of how inflammation-driven/derived chemokines may modify platelet function and concomitantly contribute to the development and or progression of complications in preeclampsia.

The plasma level of proinflammatory cytokine tumor necrosis factor-α (TNFα) was significantly higher in preeclampsia participants (Figure 2). Besides sustaining inflammation, studies investigating thrombosis in an inflammatory milieu showed that TNFα amplified collagen-mediated platelet activation, aggregation, and in vivo thrombosis [98,99]. The in vivo prothrombotic effects of TNFα required the endothelial expression of TNFα receptor-2 [98] and were dependent on enhanced endothelial production of reactive oxygen species, as well as an upregulation of P-selectin, tissue factor, and plasminogen activator inhibitor-1 expression [98]. Whether or not TNFα driven prothrombotic mechanisms are operative in hypertensive disorders of pregnancies is debatable [100,101]. Additionally, we identified other plasma cytokines elevated in PE, such as CCL1 and C-Kit/SCF cytokines, whose effects on platelet function are yet to be established (Figure 2). The increase in these cytokines’ levels is unlikely to be solely from increased platelet secretion alone. Our measurement of these plasma cytokines includes the contribution of other cells, such as the endothelial cells, also known to be dysfunctional in preeclampsia [15,16,17,18,19]. We, therefore, suggest that these cytokines are biomarkers and procoagulant agonists that need to be explored further for potential thrombo-inflammatory roles in preeclampsia.

In this study, platelet leucine-rich repeat-containing protein 27 and 42 (LRRC27/42) subunits of volume-regulated anion channels (VRAC) were markedly overexpressed in preeclampsia (Figure 3). Transcripts of LRRC27/42 have been identified in human and mouse platelets [96]; however, there is a scarcity of data on the relevance of these proteins to thrombo-inflammation in GH or PE states. In addition, our STRING analysis did not identify a plausible interaction between LRRC27/42 and the proteins enriched in PE, suggesting that there is still minimal information known about their role in PE (Figure 5). Nevertheless, it was recently shown that protein 8A (LRRC8A), another subunit of VRAC, enhanced beta-cell glucose sensing and insulin secretion [102]. In addition, we showed that the facultative glucose transporter-3 (GLUT3) was overexpressed in PE [26]; if LRRC27/42 functioned similarly as LRRC8A, both may operate in tandem with GLUT3 to enhance ‘resting state’ glucose entry in platelets and contribute to the prothrombotic tendency of preeclampsia. However, further research is needed to verify this hypothesis. In addition, our STRING analysis (Figure 5) revealed the potential for interaction between glucose transporter GLUT1 and GLUT3 (SLC2A1, SLC2A3) and inflammatory mediators enriched in the plasma of preeclampsia patients. 

Using an unbiased metabolomics approach, we identified 10 differentially expressed metabolites (Figure 6A,B). We identified allantoin, L-glutamine and L-histidine to be significantly elevated in PE when compared to healthy pregnancies (Figure 6B,C). L-glutamine was differentially expressed between GH and PE; therefore, it could potentially become a biomarker if tested in a larger number of patients. Interestingly, another study demonstrated an elevation of glutamine in PE, but no analysis of GH patients was performed [67]. Interestingly, six metabolites were significantly elevated in non-pregnant patients as compared to all three groups of pregnant patients, suggesting a shift in metabolism during pregnancy independent of prothrombotic or proinflammatory tendencies; this, too, is a subject for further investigation. A study by Austdal et al. [103] evaluated the potential of metabolomics to predict preeclampsia and gestational hypertension from urine and serum samples in early pregnancy. The authors identified 30 metabolites in serum; decreased levels of glucose, lactate and alanine were considered important for the prediction of hypertensive disorders of pregnancy. In comparison, we have examined plasma levels of metabolites during late pregnancy, and levels of L-alanine were unchanged between healthy and hypertensive complications of pregnancies. However, we identified a pathway enrichment for alanine, aspartate, and glutamate metabolism in PE and GH (Figure 6E). Thus, changes in plasma levels of these metabolites may be markers of onset and progression of hypertensive complications of pregnancies. Consistent with our findings (Figure 6B,C), Harville et al. [104] examined first-trimester serum specimens from 51 cases of hypertensive disorders of pregnancy, identified an enrichment for L-Arginine metabolism, and associated perturbations of the aminoacyl-tRNA biosynthesis pathway with hypertensive disorders of pregnancy. Notably, similar findings have been reported in studies of late-onset preeclampsia [105]. Another metabolomics study collected serum from 67 PE patients and 500 healthy pregnancies to predict metabolites significantly changing during the first trimester of PE pregnancy. The authors reported that taurine, in combination with prior risk and mean arterial blood pressure, was predictive for women who later developed PE [106,107]. In our study, taurine was not associated or elevated in preeclampsia or gestational hypertension. The relatively lower sample size of the present study may account for this lack of association.

In this study, we identified several novel proteins downregulated in hypertensive complications of pregnancy when compared to a healthy pregnancy. These include XIRP2, CCDC144A, IGHG4, CPN2, TTC6, TRAP1, GC, ZNF648, TMTC3, and CHMP4A; together, these suggest that aside from thrombosis and inflammation, other events contribute to preeclampsia onset and progression. In addition, we confirmed the findings of several studies that demonstrate the enrichment of fibrinogen and fibronectin in maternal blood during pregnancy. We also indicated fibronectin as a significant driver of hypercoagulation in preeclampsia, and that increasing plasma fibronectin levels may definitively identify pregnant patients at risk of preeclampsia. Our identification of increased expression of LRRC27/42 and S100A8/9 in platelets of preeclampsia is also novel. Nevertheless, these proteins are largely uncharacterized in pregnancy, so further validation and functional significance studies are needed. Our identification in hypertensive complications of pregnancy of increases in plasma ITIH2, ITIH3, L-glutamate, L-histidine and taurine is confirmatory of previous findings and controversial; still, it provides some insights into the mechanistic similarities between gestational hypertension and preeclampsia.

We hypothesize that placental release of vasoactive agents mediates maternal vascular endothelial damage, leading to the release into plasma of von Willebrand factors, fibronectin, and inflammatory mediators [108]. Fibronectin and procoagulant inflammatory mediators such as CXCL12 may directly induce platelet degranulation, phosphatidylserine exposure and localized thrombin formation, causing platelets in PE to microaggregate and circulate in a more extensively activated state than in a healthy pregnancy. Additionally, activated platelets and leucocytes release inflammatory mediators that further alter platelet function and perpetuate the thrombo-inflammations of preeclampsia. Therefore, increased fibronectin may be an early marker differentiating this process and PE from GH. Importantly, our discovery-size cohort analysis is the starting point for better characterizing plasma and platelet-specific proteome and metabolite changes. In conclusion, platelet activation in preeclampsia is not a bystander effect of the inflammatory milieu generated by placental dysfunction. Instead, it contributes to the thrombo-inflammation state and progression of the disorder. Therefore, the platelet activation process itself may provide several biomarkers for monitoring preeclampsia onset and progression.

## Figures and Tables

**Table 1:** Demographics, Anthropometrics and Platelet Indices in Study Participants.

	Non-Pregnant (*n* = 4) (NP)	Healthy Pregnant (*n* = 5) (PC)	Gestational Hypertension (*n* = 4) (GH)	Preeclamptic Pregnancy (*n* = 4) (PE)
**Demographics**				
Age (years)	35.0 ± 6.5	32.0 ± 7.1	35.0 ± 5.9	32.0 ± 6.1
Gestational Age (days)		275.4 ± 4.5	250.8 ± 26.5	219.6 ± 39.7
Height (cm)	162.0 ± 3.7	164.4 ± 2.8	162 ± 4.8	161.3 ± 9.8
Weight (kg)	55.8 ± 3.8	73.4 ± 9.3	84.2 ± 6.3	77.4 ± 10.3
BMI (kg/m^2^)	21.2 ± 1.6	27.9 ± 3.4	32.2 ± 4.2	29.9 ± 4.7
Urine Protein (g/L)	Not Collected	Not Collected	0.11 ± 0.04	0.60 ± 0.85
sBP (mmHg) *	105 ± 5	112 ± 11	143 ± 2	149 ± 10
dBP (mmHg) *	5 ± 4	67 ± 9	85 ± 7	90 ± 3
Hemoglobin levels (g/L)	133.3 ± 12.0	133.2 ± 7.3	120.2 ± 6.2	123.4 ± 11.1
Positive GDM Screen **	N/A	0	0	0
**Platelet Indices**				
Platelet count (10^9^/L)	270.3 ± 63.6	186.0 ± 44.6	206.8 ± 33.0	252.0 ± 65.9
Mean Platelet Volume (fL)	10.0 ± 0.8	9.1 ± 4.1	12.2 ± 0.9	11.7 ± 0.7
Immature Platelet Fraction (%)	2.4 ± 1.2	5.0 ± 3.0	9.5 ± 3.5	7.8 ± 3.1

Measurement of variables was at the time of sample collection. Data is presented as mean ± standard deviation. **Inclusion Criteria:** (1) Healthy non-pregnant females; (2) healthy gravid females presenting between 24 and 42 weeks gestational age (GA); (3) females with gestational hypertension * presenting between 24 and 42 weeks GA; (4) females with preeclampsia * presenting between 24 and 42 weeks GA.* Blood pressure (BP) cutoffs for hypertensive disorders of pregnancy: systolic blood pressure (sBP) ≥ 140 mmHg and/or diastolic blood pressure (dBP) ≥ 90 mmHg defined according to the Society of Obstetricians and Gynecologists of Canada guideline by Magee et al. (2014) (1) **Exclusion Criteria:** Females, (1) with significant pre-existing cardiovascular, respiratory, neurological, gastrointestinal, endocrine, or hematologic co-morbidities; (2) with pre-existing thrombophilia or coagulation defects; (3) receiving DVT prophylaxis.* Defined according to the Society of Obstetricians and Gynecologists of Canada guideline by Magee et al. (2014) [1]. ** GDM was determined by standard 50 g oral glucose challenge ±75 g oral glucose challenge between 24- and 28-weeks gestational age as per the Society of Obstetricians and Gynecologists of Canada guidelines by Berger et al. (2019) [38]. BMI: Body Mass Index; GDM: Gestational Diabetes Mellitus. The normal range for platelet count in women in Alberta, Canada as determined from provincial clinical records is 150–400 × 10^9^/L.

## Data Availability

The proteomics data are publicly available and were deposited in PRIDE Archives, accession number: PXD031278 (https://www.ebi.ac.uk/pride/archive/projects/PXD031278 (accessed on 24 February 2022)).

## Supplementary Materials

The following supporting information can be downloaded at: https://www.mdpi.com/article/10.3390/cells11081256/s1, Figure S1: Heatmap of proteins identified in the plasma; Table S1: TMT labelling scheme; Table S2: Peptide spectral matches (PSMs) from plasma shotgun proteomics; Table S3: Raw protein quantification from plasma proteomics; Table S4: Normalized proteins from plasma proteomics; S5: Peptide spectral matches (PSMs) from platelet shotgun proteomics; Table S6: Raw protein quantification from platelet proteomics; Table S7: Normalized proteins from platelet proteomics; Table S8: Plasma metabolomics.
