# Supplementary material for: Proteomics and Metabolomics Profiling of Platelets and Plasma Mediators of Thrombo-Inflammation in Gestational Hypertension and Preeclampsia
Source: Cells. 2022 Apr 7;11(8):1256. doi: 10.3390/cells11081256 (PMC9027992; doi:10.3390/cells11081256)
Supplement: Supplementary file 1 [file cells-11-01256-s001.zip › Figure S1.pdf]

Figure S1

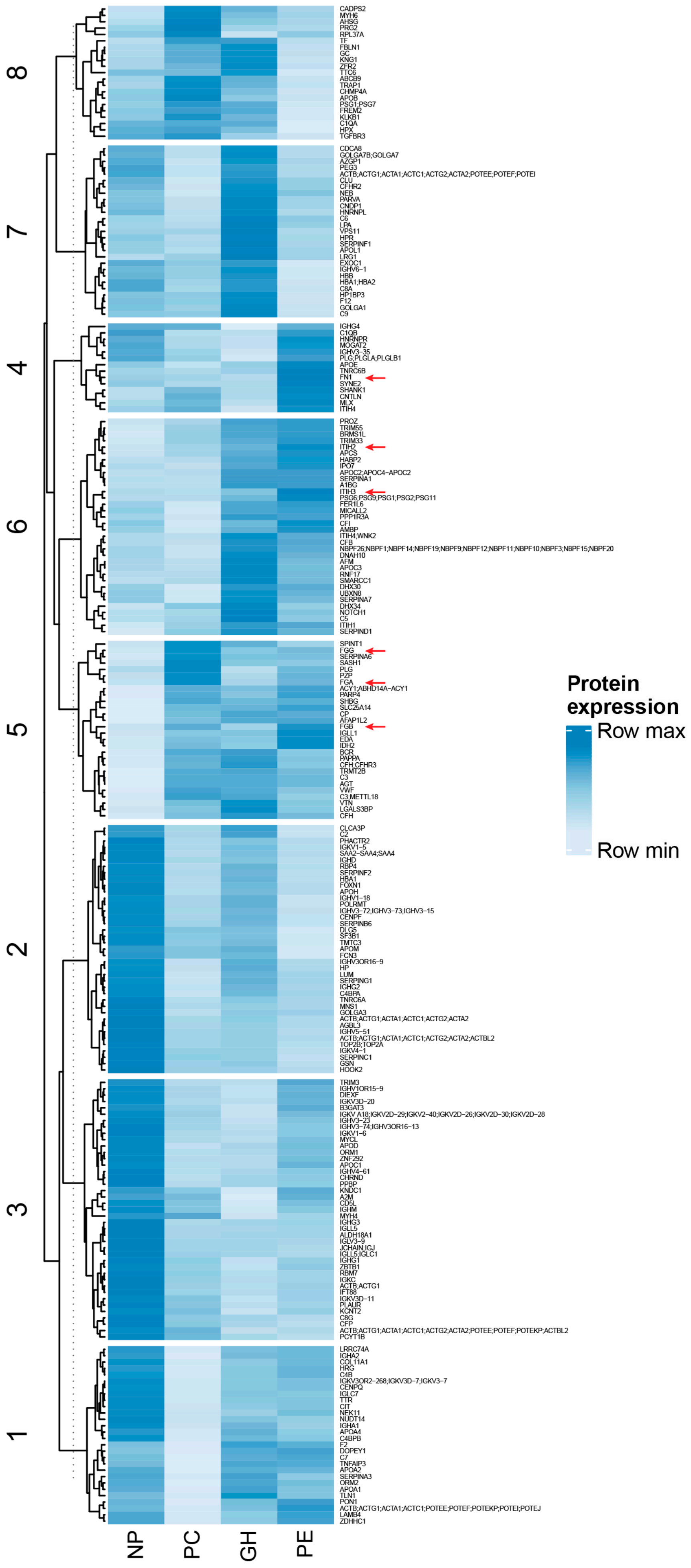

Figure S1 : Heatmap of proteins identified in the plasma. Intensity values were scaled and centered to show relative expression. The quantification was summarized per group using the median value. Proteins with missing values were removed.
